# Solving Familiar Problems: Leveraging Environmental Testing Methods for Nanomaterials to Evaluate Microplastics and Nanoplastics

**DOI:** 10.3390/nano12081332

**Published:** 2022-04-13

**Authors:** Elijah Joel Petersen, Alan James Kennedy, Thorsten Hüffer, Frank von der Kammer

**Affiliations:** 1 Biosystems and Biomaterials Division, Material Measurement Laboratory, National Institute of Standards and Technology, Gaithersburg, MD 20899, USA; 2 Environmental Laboratory, US Army Engineer Research and Development Center, Vicksburg, MS 39180, USA; alan.j.kennedy@usace.army.mil; 3 Macromolecules Innovation Institute, Virginia Polytechnic Institute and State University, Blacksburg, VA 24061, USA; 4 Department of Environmental Geosciences (EDGE), Centre for Microbiology and Environmental Systems Science, University of Vienna, 1090 Vienna, Austria; thorsten.hueffer@univie.ac.at (T.H.); frank.von.der.kammer@univie.ac.at (F.v.d.K.); 5 Research Platform Plastics in the Environment and Society (PLENTY), University of Vienna, 1090 Vienna, Austria

## 1. Introduction

The potential environmental and human health risks from microplastic (1 µm to 1 mm) and nanoplastic (<1 µm) particles (MNPs) is receiving increasing attention from scientists and the public [1,2,3]. Most particles in the environment are likely secondary particles formed from the degradation and weathering of larger pieces of plastic [4,5]. These plastic particles have a large diversity of characteristics (e.g., size, density, shape, chemical composition, additives and degree of weathering) [6].

Currently, MNP environmental fate and hazard studies use a wide range of non-standardized methods, resulting in the low comparability of results. This hinders the generation of consistent and reliable hazard data, increases the uncertainty of risk determinations and limits the use of computational models. Examples of conflicting results in the literature include some studies suggesting that MNPs pose a serious ecotoxicological risk [7,8], while other studies report minimal toxicity after the removal of additives used in polymer processing or surfactants and antimicrobials added to MNP suspensions [9,10].

**Figure 1:**
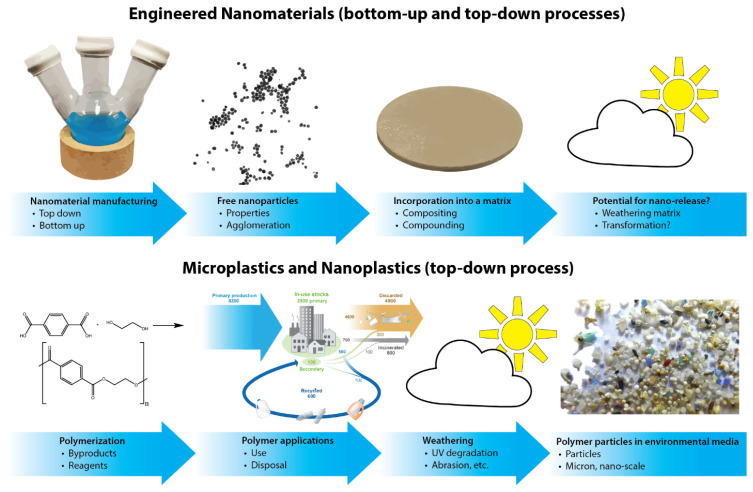
Particle generation of engineered nanomaterials (top) or microplastics and nanoplastics (bottom). Part of the figure (polymer applications) is adapted from reference [13].

Clearly, there is need for improved quality control in researching the environmental hazards of MNPs. One approach to resolve discrepancies is using existing standardized test methods. These methods were designed for dissolved substances and to avoid physical effects from particles [11]. However, MNPs at elevated concentrations could cause physical effects on organisms. This situation is similar to that confronted in research over the last decade studying the environmental behavior and toxicity of engineered nanomaterials (ENMs), where early publications also resulted in conflicting results. Given the particulate nature of both MNPs and ENMs (Figure 1), many concepts developed for the environmental risk assessment of ENMs may be adapted to improve MNP fate and hazard evaluations [12].

**Figure 2:**
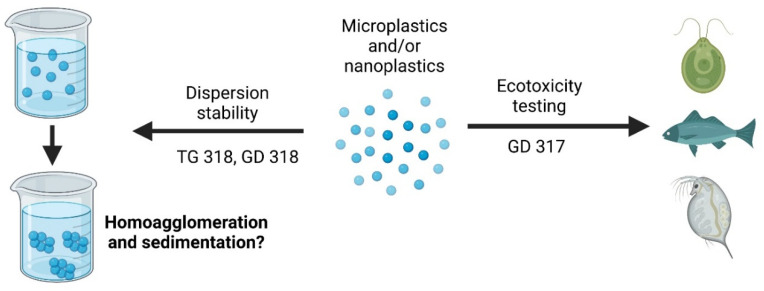
Schematic showing the potential use of GD 317 and 318 and TG 318 with nanoplastics or microplastics. Produced using biorender.org.

To improve the quality of the MNP data generated, a strategy may be to leverage OECD guidance documents (GD) (317 [14] and 318 [15]) and test guideline (TG) 318 [16], developed for ENMs (Figure 2). Many of the issues in designing ENM-specific test improvements are applicable to MNPs. However, there are MNP-specific considerations that may require alterations to the methods for ENMs. Questions about the regulatory applicability of results obtained using these methods for incidental particles is beyond the scope of this manuscript.

## 2. Applicability of and Key Lessons from OECD GD 317

Generally, the approaches described in OECD GD 317 [14] for ENMs are applicable for the ecotoxicity testing of MNPs to accommodate consistent and reliable testing. For example, many OECD pelagic organism toxicity test guidelines specify that the exposure concentration should remain within 20% of the initial concentration for the entire testing duration (or between water renewals) [11,14,17]. For unstable MNP dispersions, OECD GD 317 provides extensive guidance on this topic: preliminary suspension stability screening experiments, a hierarchy of potential test media manipulations (e.g., pH, ionic strength) to improve stability, more frequent test media renewals, and the use of time-weighted averages to better represent dynamic exposures. Some microplastics may settle out of dispersion even without agglomeration, independent of media composition. Mild agitation, when possible, can counter the effect of sedimentation.

OECD GD 317 also provides recommendations about control measurements that can be performed to better understand the assay results and avoid artifacts. For example, it may be important for assays that measure absorbance or fluorescence to test if the particles present could have a similar signal to the measurand [18,19]. This control is important for cell-based measurements (e.g., algae [20], fish cells [21]). Depending upon the study goals, it may be important to determine if the toxicity from a suspension is caused by the particles, or dissolved substances such as leached additives, dispersants, or antimicrobials.

A key question for conducting pelagic aquatic toxicity tests is the study design and whether to test (1) only dissolved constituents leached from the plastics, (2) dissolved species and suspended particles, or (3) suspended particles, settled particles, and dissolved species. For MNPs, dissolution may be less prevalent but a solubilized component may be realized through the weathering and leaching of polymers, including additives (such as lead or phthalates) [22], plasticizers, and unpolymerized monomers.

## 3. Applicability of and Key Lessons from OECD GD 318 and TG 318

Since these documents deal with the specific behavior of ENMs in environmental media and focus on inorganic ENMs, it is important to assess how this translates to MNPs. The homo- and heteroagglomeration of MNPs is as important for their transport behavior as for ENMs. The determination of the relevant parameters can—in principle—follow the experimental approaches described in GD 318 and TG 318, with a few considerations.

The media composition for agglomeration testing in TG/GD 318 is suitable for the testing of MNPs. However, the test requires (I) a particle density of >1 g*cm^−3^, which would exclude some low-density polymers, and (II) that a settling of unagglomerated particles does not occur, which would exclude particles larger than ~2 µm at densities >1.2 g*cm^−3^. Calculating the settling velocity of the MNPs under investigation is advised. The size distribution needs to be considered to avoid losses of unagglomerated larger particles. Another challenge is the analysis of the remaining MNP mass concentration in the supernatant, which should be performed with established techniques for the quantification of the MNP under investigation. Furthermore, the size of MNPs needs to be evaluated for the suitability of heteroagglomeration testing following GD 318. The floc size of natural suspended particulate matter is approximately 5 µm to 50 µm, and sewage sludge flocs may reach 100 µm or more. When testing nanoplastics, the plastic particles will be incorporated into flocs (similar to ENMs) and the unagglomerated plastic particles should not be removed in the separation step (centrifugation or settling). For microplastics, a well-defined separation of floc-associated and free microplastics may be difficult. Separation can be hindered by flocs adsorbing onto particles when they are larger than the flocs, and from free microplastics having a similar sedimentation behavior to the flocs and floc-associated microplastics.

The “dissolution” test for MNPs could be used to test for the leaching of additives from MNPs. However, the heterogeneity in materials, particle sizes and target substances (e.g., leaching of organic chemicals compared with dissolving metals) will require different experimental and analytical approaches. To start this discussion now will enable researchers to consider the leaching from MNPs in the development of dissolution testing protocols for ENMs.

## 4. Overarching Issues for Both Environmental Hazard and Fate Testing

Preparing a stable suspension requires careful consideration. The use of ultrasonication may not be applicable for MNPs if this would cause the disintegration of the plastic particles, increased leaching of additives, or some other unintended changes to the particles, such as modifications to the surface chemistry. Low-density plastics or highly hydrophobic plastics may become entrained in surface tension and float. For these particles, many OECD TGs may be less applicable.

The mass concentration was recommended as the default metric for testing with ENMs in OECD GD 317 and TG 318 [23]. This contrasts with the literature for microplastics where the most commonly used dose metric is the particle number concentration [24]. The analytical approaches most often used for microplastic particles will not be feasible for nanoplastic particles [2], and particle dosimetry to monitor the exposure concentration may be challenging at lower concentrations. While it is unclear what dose metric to recommend for MNP testing, the continued development and standardization of analytical methods remains a key need.

Unlike for ENMs, there is currently a lack of MNP reference materials, except for monodisperse polystyrene spheres; these spheres are frequently used in nanoplastic studies and have limited environmental relevance. This lack of environmentally relevant reference materials hinders interlaboratory analytical method evaluation and ensuring the comparability among studies enabled by testing the same materials.

Another challenge is determining what plastic particles to test. While ENMs are often tested “as produced,” plastics typically require weathering in the environment prior to being broken down into microplastics or nanoplastics. Standardized methods are needed to produce environmentally relevant materials. It is important to note that it is substantially more challenging to characterize heterogenous mixtures of plastic particles (different sizes, plastic formulations, shapes, extent of weathering, polymer chain degradation, etc.) than particles of a single composition and primary particle size, as is typical for ENMs. Characterizing the suspended concentration is also more complex with heterogeneous mixtures. For example, the sedimentation of larger particles could have a substantial effect on the mass concentration, but not the particle number concentration.

In summary, the use of the recent OECD TG and GDs developed for ENMs can speed improvements to the reproducibility of the results obtained in MNP studies and support meta-analyses. By having more robust methods, conflicting results among studies can be minimized and a more accurate picture of the potential environmental risks of MNPs can be obtained.
